# Leaf Economics and Local Adaptation: Genetic and Plastic Responses Within Mediterranean Annual Species to Macro‐ and Microclimatic Aridity Gradients

**DOI:** 10.1002/ece3.73277

**Published:** 2026-03-17

**Authors:** Ruchi Tiwari, Francine Hellmanzik, Florian Gade, Johannes Metz

**Affiliations:** ^1^ Plant Ecology & Nature Conservation Group, Institute of Biology & Chemistry, University of Hildesheim Hildesheim Germany

**Keywords:** competition, drought adaptation, leaf dry matter content, leaf economics Spectrum, local adaptation, macroclimate and microclimate, Mediterranean annual plants, phenotypic plasticity, rainfall gradient, specific leaf area

## Abstract

1. Understanding how plants adjust to changing rainfall and intensified drought is vital, particularly in the Mediterranean region. The Leaf Economics Spectrum (LES) framework distinguishes fast‐growing plants with high specific leaf area (SLA) and low leaf dry matter content (LDMC) from resource‐conserving plants with opposite traits. In drier environments, plants can be expected to exhibit lower SLA and higher LDMC to minimize water loss and enhance drought resilience. However, it is unclear whether this expectation holds within species among locally adapted populations, and how it is modulated plastically by drought and competition.
2. This study investigated SLA and LDMC in five Mediterranean annual plant species across a macroclimatic rainfall gradient (89–926 mm annual rainfall) and contrasting microclimates: more mesic north‐ and more arid south exposures. Seeds were collected at 15 sites along this gradient, and plants were grown under controlled greenhouse conditions with three experimental treatments: control, drought, and competition.
3. Contrary to LES expectations, plants from drier macroclimates had mostly evolved higher SLA and lower LDMC, suggesting that drought escape strategies via rapid growth and early reproduction were favored over conservative resource use. Microclimatic variation in leaf traits was smaller than macroclimatic variation, although in two species it showed the same trend, with south‐exposure plants displaying lower SLA and/or higher LDMC. Plastic responses to drought consistently reduced SLA and increased LDMC, and vice‐versa under competition, thus countering the genetically‐based macroclimatic pattern. Interestingly, mesic populations showed stronger plastic shifts to competition.
4. Our results show that evolution favored acquisitive leaves in drier sites, while plasticity modulated leaf traits in the opposite direction under drought stress and competition. These findings illustrate the interplay between evolutionary and plastic responses, underscoring the importance of considering both genetic differentiation and plasticity when assessing annual plant responses to future climate change.

## Graphical abstract

This study examined how Mediterranean annual plants adjust leaf traits (SLA and LDMC) across rainfall gradients and microclimates, considering both genetic differentiation and plasticity under drought and competition. Contrary to expectations from the Leaf Economics Spectrum, populations from drier macroclimates evolved more acquisitive leaves (higher SLA, lower LDMC), while plastic responses to drought and competition shifted traits in the opposite direction. These findings highlight the contrasting roles of evolution and plasticity in shaping plant responses to climate variability.

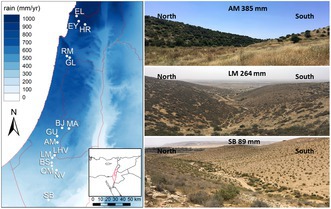

## 1 Introduction

The Leaf Economics Spectrum (LES) provides a foundational framework for understanding plant functional strategies along a continuum from fast‐growing, resource‐acquisitive plants to slow‐growing, resource‐conservative ones (Reich 2014; Wright et al. 2004). Key LES traits include specific leaf area (SLA, leaf area per unit dry mass) and leaf dry matter content (LDMC, dry mass per unit fresh mass). Plants with high SLA are associated with high photosynthetic rates and rapid growth under favorable conditions (Shipley et al. 2005; Wright et al. 2004), whereas high LDMC signals investment in durable, stress‐tolerant structures, especially under drought (Smart et al. 2017). Changes in SLA and LDMC may reflect a trade‐off between maximizing growth and competitive ability under resource‐rich conditions (high SLA, low LDMC) versus enhancing tolerance to drought stress under arid conditions (low SLA, high LDMC), echoing the antagonism of competition and stress as key drivers along many environmental gradients (Bongers et al. 2017; Janíková et al. 2024; Lorts and Lasky 2020). Congruently, global analyses consistently show that plants with low SLA and high LDMC dominate in arid and nutrient‐poor environments, supporting conservative strategies that enhance survival under stress (Han et al. 2025; Liu et al. 2023; Radford‐Smith and Dwyer 2025). These patterns are often attributed to species turnover (Wright et al. 2004). Yet, intraspecific trait variation, driven by plasticity or genetically‐based local adaptation, is increasingly recognized to shape trait responses (Fajardo and Siefert 2018; Navarro et al. 2022; Pichon et al. 2022). Still, the mechanistic role of plasticity versus local adaptation in shaping SLA and LDMC has been rarely disentangled.

Local adaptation involves genetically based, heritable trait differentiation that enhances fitness in the local environment (Kawecki and Ebert 2004; de Vries et al. 2024). Along aridity gradients, selection should favor conservative traits in drier sites such as low SLA and high LDMC due to their water‐saving and structural benefits, whereas mesic sites may select for acquisitive traits that support fast growth (Baruch et al. 2017; Dwyer et al. 2014; Grime 1974, 1977; Radford‐Smith and Dwyer 2025; Reich 2014). Indeed, perennial species from arid sites generally showed lower SLA and higher LDMC (Baruch et al. 2017; Carlson et al. 2016; Ramírez‐Valiente et al. 2010), in line with LES predictions. However, annual species often deviate from these patterns. Plants originating from drier sites tended to have lower SLA in *Chamaecrista fasciculata* (Etterson 2004) and *Arabidopsis thaliana* (Fletcher et al. 2022), higher SLA in *Plantago patagonica* (Christie et al. 2022), and showed no clear trend in several Mediterranean annuals (Bergholz et al. 2017; Kurze et al. 2017). These examples suggest that the rapid life history and drought escape strategy in annuals can lead to trait syndromes that diverge from LES predictions (Franks et al. 2007; Kooyers 2015), rendering leaf trait variation along aridity gradients less understood in annuals and calling for more studies.

While most studies on local adaptation have focused on broad macroclimatic gradients, exposure aspect can create sharp microclimatic contrasts over short distances that mirror large‐scale climatic patterns (Saeidi et al. 2023). In the Northern Hemisphere, south‐exposed slopes are typically warmer and drier due to greater solar radiation, carry sparser vegetation, and higher abundances of drought‐adapted species than their north‐facing counterparts (Blanco‐Sánchez et al. 2022; Kutiel and Lavee 1999; Nevo 2012; Pan et al. 2023). These conditions may foster localized trait differentiation and act as natural laboratories for studying microclimatic adaptation (Estevo et al. 2022; Nevo 2012). Indeed, conspecifics from north‐exposed slopes at 15 sites showed greater competitive ability, while those from south exposures coped partly better with drought (Gade and Metz 2024). Also, studies from single sites reported some genotypic or phenotypic differences between north‐ and south‐facing conspecifics (Nevo 2012; Qian et al. 2018; Wang et al. 2023). However, it is unclear whether conspecifics from opposite exposures evolved differential SLA and LDMC that contribute to microclimatic local adaptation. While alpine meadow communities in Tibet showed overall more conservative leaf traits (lower SLA, higher LDMC) at south exposures (Pan et al. 2023), selection favored acquisitive leaf traits on both exposures in two Mediterranean shrubs in contrast to LES expectation (Blanco‐Sánchez et al. 2022; Carlucci et al. 2015). The only study reporting leaf traits for microclimatic local adaptation between north‐ and south exposures found no difference in SLA and LDMC in annual *Brachypodium hybridum* (Kurze et al. 2017).

Phenotypic plasticity, the ability of a genotype to modify traits in response to environmental conditions without genetic change, is a key mechanism for coping with variability in resources such as water (Sommer 2020; Stamp and Hadfield 2020). Experimental studies manipulating water availability through controlled watering or reciprocal transplant designs generally found that drought reduces SLA and increases LDMC, favoring conservative traits that limit water loss and prolong leaf lifespan (Etterson 2004; Kramp et al. 2022; Oyanoghafo et al. 2023; Ramos‐Muñoz et al. 2024). Similarly, grassland species reduce SLA and increase LDMC in drier years, reflecting phenotypic plasticity in response to interannual variation in water availability (Wheeler et al. 2023). However, some studies have reported the opposite pattern. O'Hara et al. (2016), found that SLA increased under drought in *Brassica rapa* , both plastically in greenhouse experiments and evolutionarily over seven generations in a resurrection experiment. Competition may also change SLA and LDMC plastically, but empirical evidence has remained scarce and heterogeneous. Higher species richness (putatively increasing light competition) increased SLA in temperate forbs (Lipowsky et al. 2015). In subtropical tree saplings, interspecific competition similarly increased SLA for light capture, but also increased LDMC for tougher leaves (Yang et al. 2021). In temperate Carex species, competition decreased SLA and increased LDMC, thus favoring conservative leaves (Janíková et al. 2024). Seasonal dynamics can further modulate leaf plasticity, as well as interacting effects of drought and competition (Hao et al. 2023; Janíková et al. 2024; Lorts and Lasky 2020), highlighting the need for further studies on plasticity in response to drought and competition.

This study investigated local adaptation across combined macro‐ and microclimatic aridity gradients by isolating genetic variation in SLA and LDMC, and further assessed plastic responses to drought and competition. Five Mediterranean annual species were selected, including two grasses (*Brachypodium hybridum*, *Avena sterilis* ) and three forbs (*Hedypnois rhagadioloides, Hippocrepis unisiliquosa, Anagallis arvensis*), sampled along a natural macroclimatic rainfall gradient in Israel ranging from 89 to 926 mm/yr. At each of 15 sites, seeds were collected from both north‐ and south exposures to capture microclimatic variation. Offspring were raised under common greenhouse conditions to isolate genetic effects on leaf traits. For two focal species (*Brachypodium hybridum, Hedypnois rhagadioloides*), plastic responses to drought and competition treatments were evaluated. We tested whether plants from arid sites and south exposures evolved lower SLA and higher LDMC, reflecting conservative leaf strategies. We further tested for corresponding plastic responses, that is, whether SLA decreases and LDMC increases under drought, but the opposite occurs under competition, and whether these responses depend on macro‐ and microclimatic origin.

## 2 Materials and Methods

### 2.1 Sampling Sites

**FIGURE 1:**
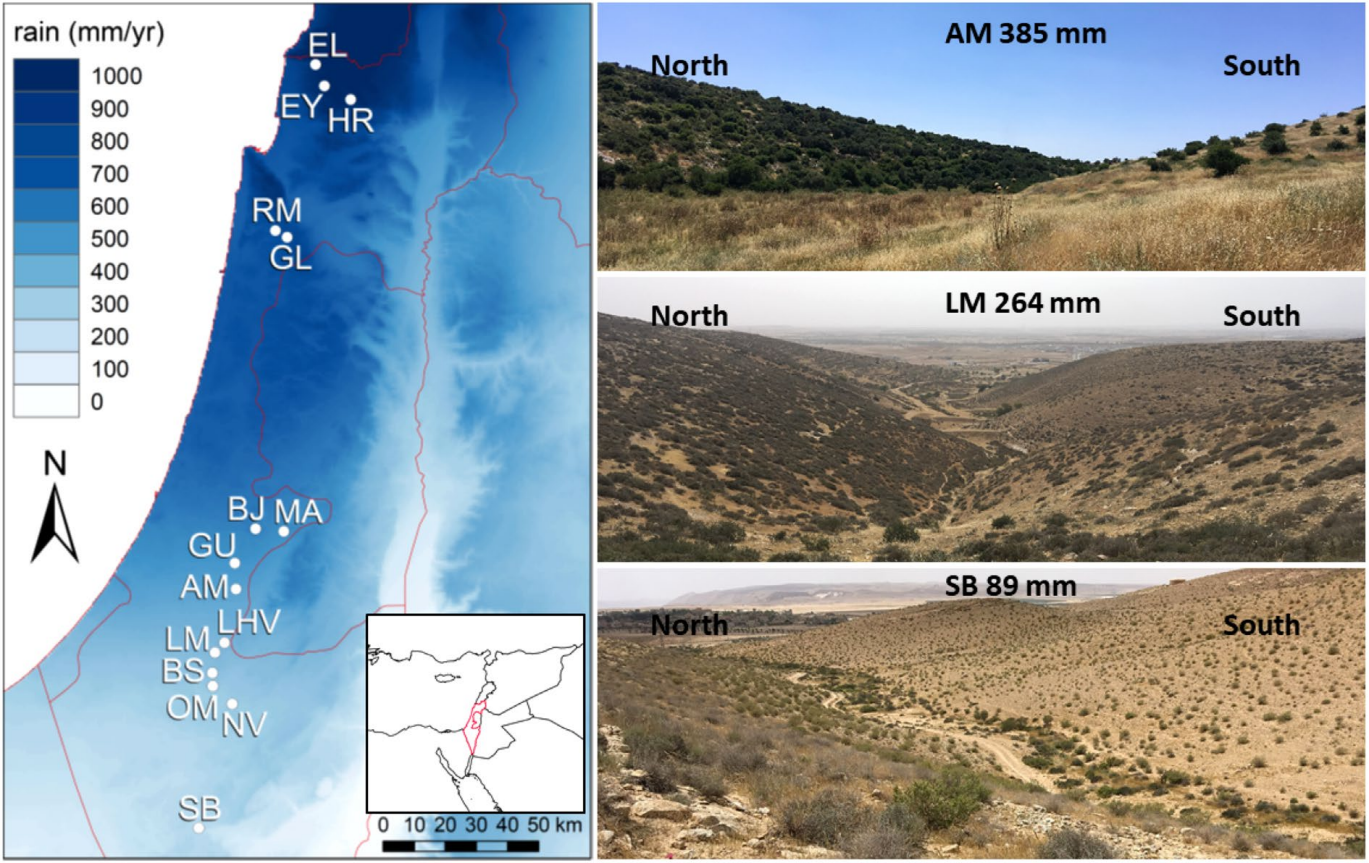
Study region and climatic context. Left: Mean annual rainfall of the study region showing the locations of the 15 sampling sites along a macroclimatic rainfall gradient in Israel, alongside (inset map) its broader geographic setting and macroclimatic context along the Mediterranean–desert transition zone. Right: Microclimatic contrast between north‐facing exposures (mesic conditions with denser vegetation) and south‐facing exposures (more arid conditions) in three representative sites, illustrating the sharp vegetation turnover associated with exposure aspect (photo credit: J. Metz).

The study was conducted along a macroclimatic aridity gradient in Israel, spanning approximately 250 km from north to south (Figure 1). We used the same 15 sampling sites described in Gade and Metz (2024), which range from 89 to 926 mm annual rainfall (details in Appendix Table S1). All sites share similar limestone bedrock and mean annual temperatures (17.8°C–19.8°C). Denser vegetation occurs in wetter sites (Golodets et al. 2013; Tielbörger et al. 2014; Figure 1), causing stronger competition than in drier sites (Metz and Tielbörger 2016; Schiffers and Tielbörger 2006). At each site, paired north‐ and south exposures separated by small dry valleys served as microclimatic contrasts, with south exposures being drier and more sparsely vegetated (Figure 1; Appendix Table S1).

### 2.2 Study Species

**FIGURE 2:**
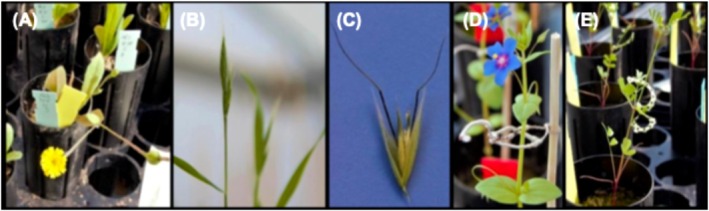
Illustrations of the five study species: (A) *Hedypnois rhagadioloides* (Asteraceae), (B) *Brachypodium hybridum* (Poaceae), (C) *Avena sterilis* (Poaceae), (D) *Anagallis arvensis* (Primulaceae), *and* (E) *Hippocrepis unisiliquosa* (Fabaceae).

We studied five winter annual species that spanned four Angiosperm families: two grass species, *Brachypodium hybridum* and *Avena sterilis* (both Poaceae), and three forb species, *Hedypnois rhagadioloides* (Asteraceae), *Hippocrepis unisiliquosa* (Fabaceae), and *Anagallis arvensis* (Primulaceae) (Figure 2). These species co‐occur frequently in the herbaceous layer of shrublands and woodlands in our study region (Feinbrun‐Dothan et al. 1998) and are predominantly autogamous, favoring local ecotypic differentiation via reduced gene flow (Boaz et al. 1990).

### 2.3 Seed Sampling and Plant Cultivation

In May 2019, seeds were collected from both north‐ and south exposures of all 15 sites. For each species, approximately 15 maternal individuals (hereafter termed genotypes) per exposure were randomly sampled within a 0.5‐ha area, maintaining a minimum distance of 3 m between sampled plants to reduce genetic relatedness. As fewer than 15 genotypes per species were available on some exposures and sites, final numbers were: 431 genotypes for *Brachypodium hybridum*, 384 for *Avena sterilis* , 371 for *Hedypnois rhagadioloides*, 334 for *Hippocrepis unisiliquosa* , and 330 for *Anagallis arvensis* .

In a first experiment, plants were cultivated under common, controlled greenhouse conditions at the University of Hildesheim, Germany, from December 2019 to May 2020. Field‐collected seeds were sown into 400 mL pots (5.7 cm diameter, 25 cm depth) filled with unfertilized silty clay soil from Hildesheim that resembled limestone‐derived soils of the study region (pH 7.2 ± 0.1, 2.9% ± 0.1% organic carbon, 56 ± 16 ppm total nitrogen; Zwikel et al. 2007). Each pot received 5–10 seeds of a single genotype, totaling 1848 pots across all species. On 19 December 2019, each pot was watered with 40 mL (equivalent to a 16 mm water column of rainfall) to initiate germination. Seedlings were thinned to one random target individual per pot post‐emergence. Germination rates were generally high, except for *Anagallis arvensis* , which showed reduced seedling emergence (Appendix Table S1).

Greenhouse temperature ranged from 15°C–20°C in winter (minimum 10°C) to 20°C–30°C in late spring (maximum 35°C) to simulate field conditions. The photoperiod was supplemented by LED greenhouse lights, starting at 10 h in December and increasing weekly by 15 min to mimic natural day length progression in Israel (Aronson et al. 1992). Pots were randomized within species and re‐randomized every 3 weeks to reduce positional effects. All plants were watered approximately weekly with 40 mL to provide uniform, benign growth conditions. Cross‐pollination was prevented among genotypes (as in Metz et al. 2020) to obtain full‐sibling seeds per genotype. Irrigation ceased 2 weeks before harvest in May 2020.

### 2.4 Experimental Treatments for *Brachypodium hybridum* and *Hedypnois rhagadioloides*

In a second experiment conducted during the following winter season (November 2020–May 2021), plastic responses to drought and competition were assessed in only two species for feasibility, one grass (*Brachypodium hybridum*) and one forb (*Hedypnois rhagadioloides*). These species were selected because they reflected contrasting functional types, germinated well under greenhouse conditions, and provided the highest genotype replication for grasses and forbs, respectively (details in Gade and Metz 2024). Three replicate pots per genotype were assigned to one of three treatments: control, drought, or competition, resulting in 1269 pots for *Brachypodium hybridum* and 948 pots for *Hedypnois rhagadioloides*. Each pot was filled with the same soil used previously and received five intact seeds of a single genotype, using the seed material derived from the first experiment under common conditions. To facilitate handling, the two species were grown on separate greenhouse tables. Pots were randomly arranged within species and re‐randomized every 3 weeks.

The experiment began on 19 November 2020, with all pots watered with 40 mL, followed by an additional 40 mL on 9 December and 20 December to ensure germination and seedling establishment. Seedlings were thinned to one random target per pot by mid‐January 2021.

The control treatment received 40 mL of water approximately weekly throughout the experiment, simulating benign growth conditions. The drought treatment mimicked low rainfall conditions typical of arid sites: pots were watered weekly on the same dates as the control treatment but received only 10 mL between 29 December and 17 February, and 15 mL thereafter to reflect increasing water demand during growth. Visible wilting occurred prior to watering events, confirming water limitation. The competition treatment received the same watering as the control (40 mL on the same dates) but included two additional competitor plants per pot: one *Plantago afra* L. (Plantaginaceae) and one *Avena sterilis* L. (Poaceae), representing common neighbors in the sampling sites. Competitor seeds originated from intermediate rainfall sites (MA, BJ). Six seeds of *Plantago afra* and three of *Avena sterilis* were sown per pot, thinned to one plant per species. In both target species, the drought treatment reduced fitness by approximately 50% and the competition treatment by approximately 70% compared to the control treatment, confirming that these experimental treatments effectively challenged plant fitness (Gade and Metz 2024).

### 2.5 Measurement of Leaf Traits

Two functional leaf traits were assessed: SLA, calculated as leaf area divided by dry mass, and LDMC, defined as dry mass divided by fresh mass, following standard protocols (Pérez‐Harguindeguy et al. 2013). Fully expanded, healthy leaves were harvested, immediately weighed (fresh mass), photographed for area measurement using ImageJ, then oven‐dried at 60°C for 24 h to determine dry mass. Leaf sampling occurred approximately during each species' flowering period. In the first experiment, *Brachypodium hybridum* was sampled between 26 February and 9 March 2020, followed by *Avena sterilis* from 10 to 19 March 2020, *Hedypnois rhagadioloides* between 23 and 26 March 2020, and *Hippocrepis unisiliquosa* between 30 March and 2 April 2020. Finally, *Anagallis arvensis* was sampled from 6 to 7 April 2020. In the second experiment, *Brachypodium hybridum* was sampled between 8 and 19 February 2021 and *Hedypnois rhagadioloides* between 1 and 12 March 2021.

### 2.6 Statistical Analysis

For the first experiment, we tested separately for all five species and both traits whether SLA and LDMC varied due to the plants' origin along the macro‐ and microclimatic aridity gradients. Linear mixed‐effects models were fitted in R 4.2.2 (R Core Team 2024) using the lme4 package (Bates et al. 2015), with mean annual rainfall (continuous) and exposure (north vs. south) as fixed effects, including their interaction. Site as a random effect accounted for the non‐independence of individuals from the same location. Fixed effects were tested using Type II Wald F‐tests with Kenward‐Roger approximated degrees of freedom via the car package (Fox and Weisberg 2019).

For the second experiment, including experimental treatments for *Brachypodium hybridum* and *Hedypnois rhagadioloides*, SLA and LDMC were analyzed using similar mixed‐effects models. Fixed effects included rainfall (continuous), exposure (categorical), and treatment (categorical: control, drought, competition), plus their interactions. Genotype nested in site was modeled as a random effect to reflect the experimental design with three replicates per genotype (one per treatment). Post hoc comparisons were conducted using the emmeans package (Lenth 2023), with the emmeans() function for treatment contrasts and emtrends() for interactions involving rainfall and treatment.

## 3 Results

### 3.1 Effects of Rainfall and Exposure on Leaf Traits

**FIGURE 3:**
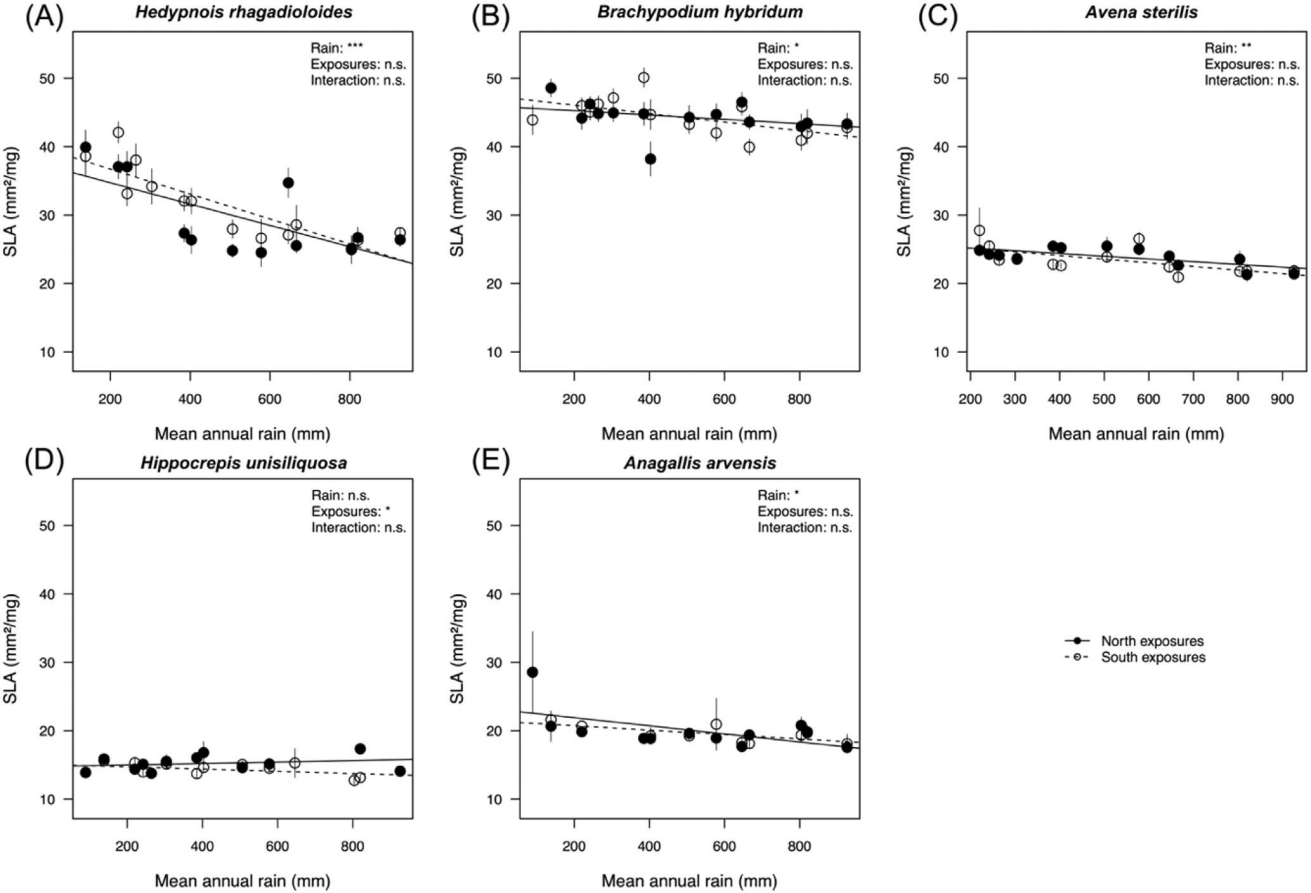
Specific leaf area (SLA) in plants originating from different sites along the macroclimatic rainfall gradient (*x*‐axes), and from either north‐facing (filled circles) or south‐facing exposures (open circles), for five study species (A—E) grown under common greenhouse conditions. Error bars represent ± SE of the mean. Significance levels are shown for Rain, Exposure, and Rain × Exposure Interaction to ease interpretation: ***p ≤ 0.001; **p ≤ 0.01; *p ≤ 0.05; ns not significant; see Table 1.

**FIGURE 4:**
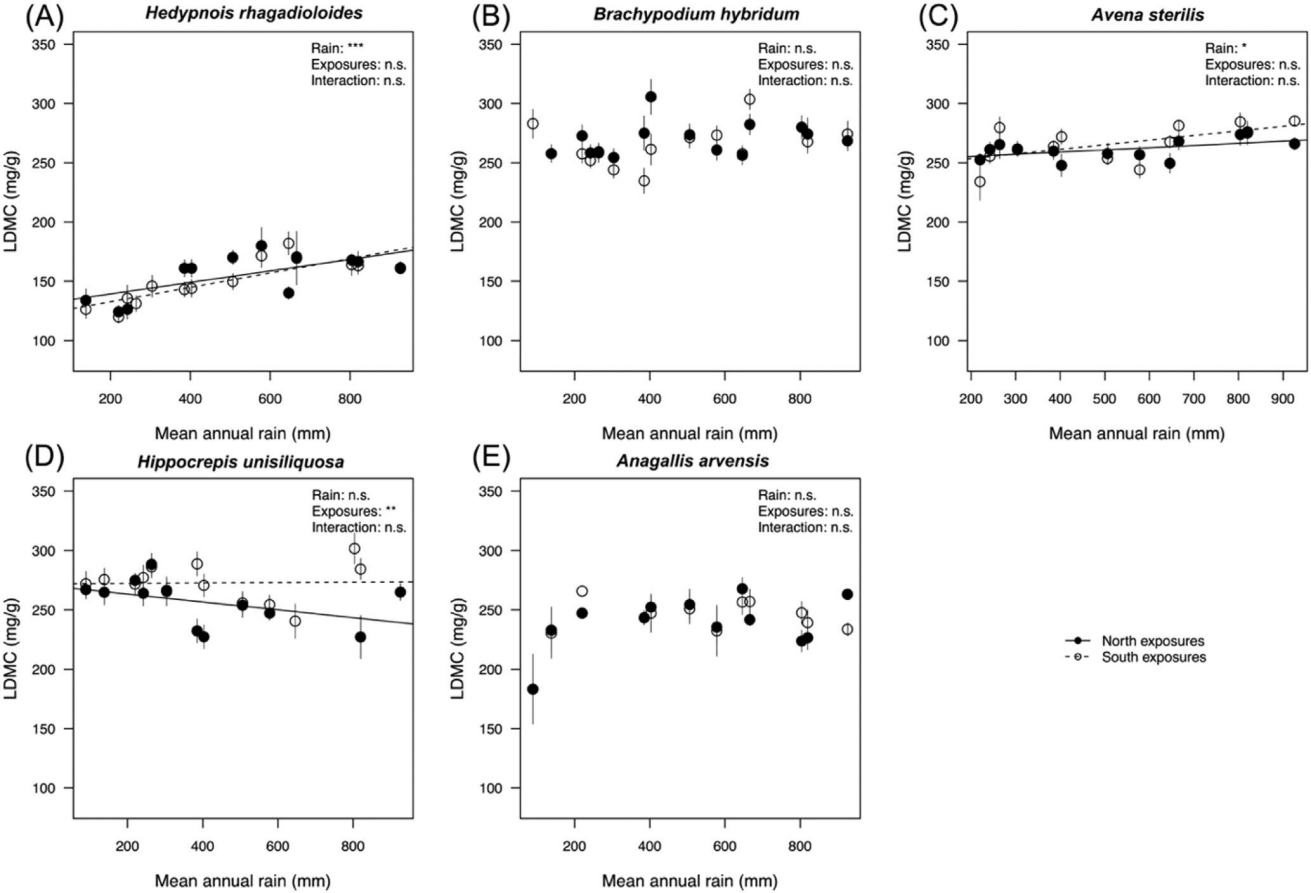
Leaf dry matter content (LDMC) in plants originating from different sites along the macroclimatic rainfall gradient (*x*‐axes), and from either north‐facing (filled circles) or south‐facing exposures (open circles), for five study species (A—E) grown under common greenhouse conditions. Error bars represent ± SE of the mean. Significance levels are shown for Rain, Exposure, and Rain × Exposure Interaction to ease interpretation: ***p ≤ 0.001; **p ≤ 0.01; *p ≤ 0.05; ns not significant; see Table 2.

Leaf traits varied widely across our study species (Table 1; Figures 3 and 4), which included two grasses (*Brachypodium hybridum*, *Avena sterilis* ) and three forbs (*Hedypnois rhagadioloides, Hippocrepis unisiliquosa*, *Anagallis arvensis* ). SLA was highest in *Brachypodium hybridum* (mean c. 44 mm^2^/mg), followed by *Hedypnois rhagadioloides* (mean c. 31), *Avena sterilis* (mean c. 24), *Anagallis arvensis* (mean c. 20), and lowest in *Hippocrepis unisiliquosa* (mean c. 15). In contrast, LDMC was highest in *Brachypodium hybridum* and *Hippocrepis unisiliquosa* (both mean c. 268 mg/g), followed by *Avena sterilis* (mean c. 263), *Anagallis arvensis* (mean c. 244), and lowest in *Hedypnois rhagadioloides* (mean c. 152). Among species, *Hedypnois rhagadioloides* had the most acquisitive leaves (high SLA, lowest LDMC), *Hippocrepis unisiliquosa* the most conservative leaves (lowest SLA, highest LDMC). Although trait values varied among species, no difference was apparent between grasses and forbs as functional groups, indicating that differences were rather species‐specific.

**TABLE 1:** Results of linear mixed models testing the response of SLA to annual rainfall at origin along the macroclimatic gradient (rain), exposure (north vs. south) in five annual species. Degrees of freedom (df) were approximated via Kenward–Roger. Bold indicates significant *p*‐values (*p* < 0.05).

Species		Df, Df res	F	*p*
*Hedypnois rhagadioloides*	Rain	1, 11.3	28.39	**0.0002**
	Exposure	1, 311.6	0.95	0.3309
	Rain × Exposure	1, 311.0	0.69	0.4066
*Brachypodium hybridum*	Rain	1, 13.8	7.41	**0.0166**
	Exposure	1, 418.9	0.40	0.5265
	Rain × Exposure	1, 375.8	2.64	0.1052
*Avena sterilis*	Rain	1, 11.9	11.77	**0.0051**
	Exposure	1, 359.9	3.36	0.0676
	Rain × Exposure	1, 364.0	1.01	0.3164
*Hippocrepis unisiliquosa*	Rain	1, 14.3	1.78	0.2030
	Exposure	1, 319.2	4.11	**0.0435**
	Rain × Exposure	1, 127.5	2.18	0.1422
*Anagallis arvensis*	Rain	1, 11.6	5.87	**0.0328**
	Exposure	1, 104.1	0.04	0.8429
	Rain × Exposure	1, 108.8	0.02	0.8966

**FIGURE 5:**
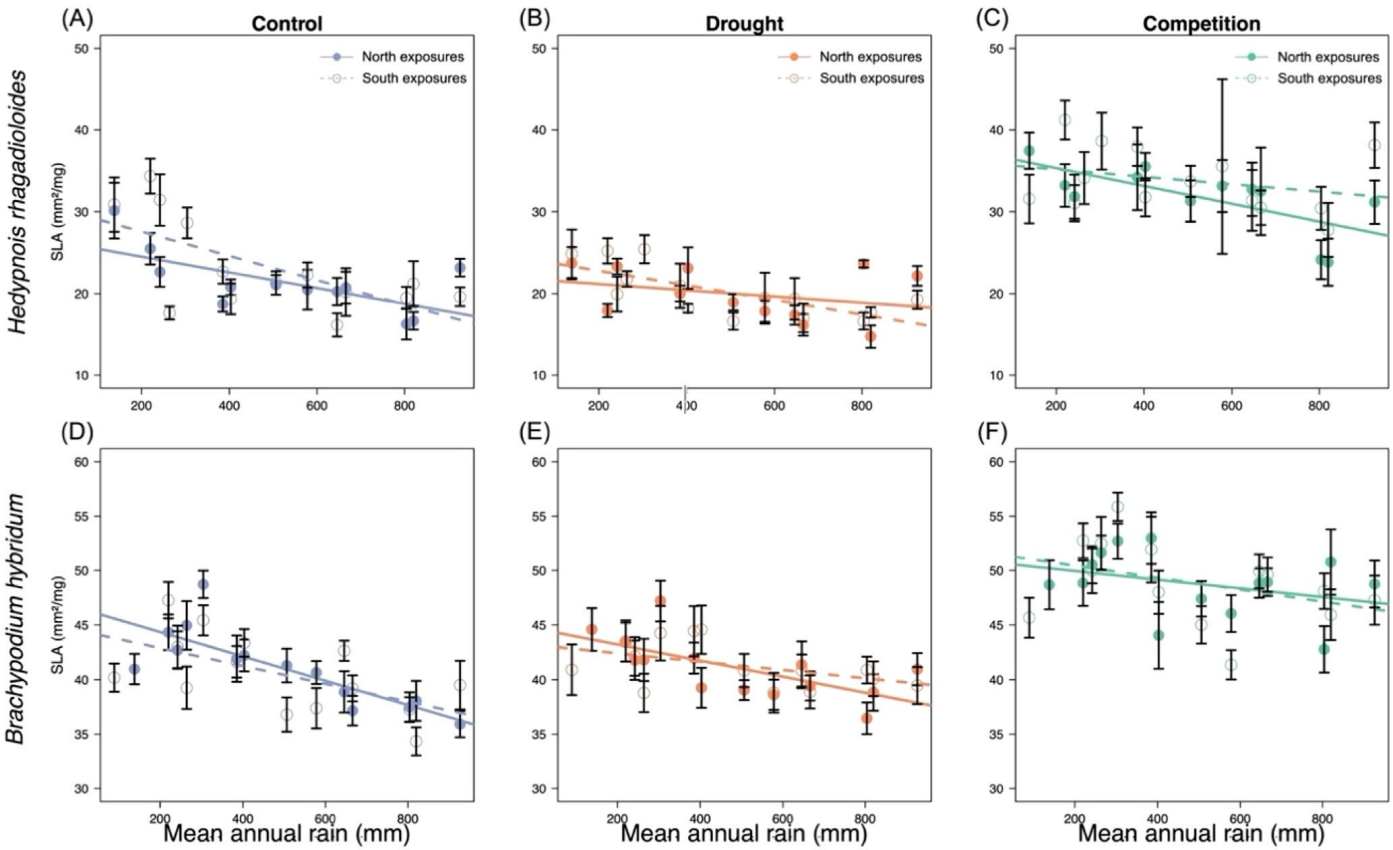
Specific leaf area (SLA) in two plant species grown under three greenhouse treatments: Control (A, D; in blue), drought stress (B, E; in orange), and competition (C, F; in green). Plants originated from different sites across the macroclimatic rainfall gradient (*x*‐axes), and from either north‐facing (filled circles) or south‐facing exposures (open circles). Error bars represent ± SE of the mean. Statistical results are provided in Table 3.

**FIGURE 6:**
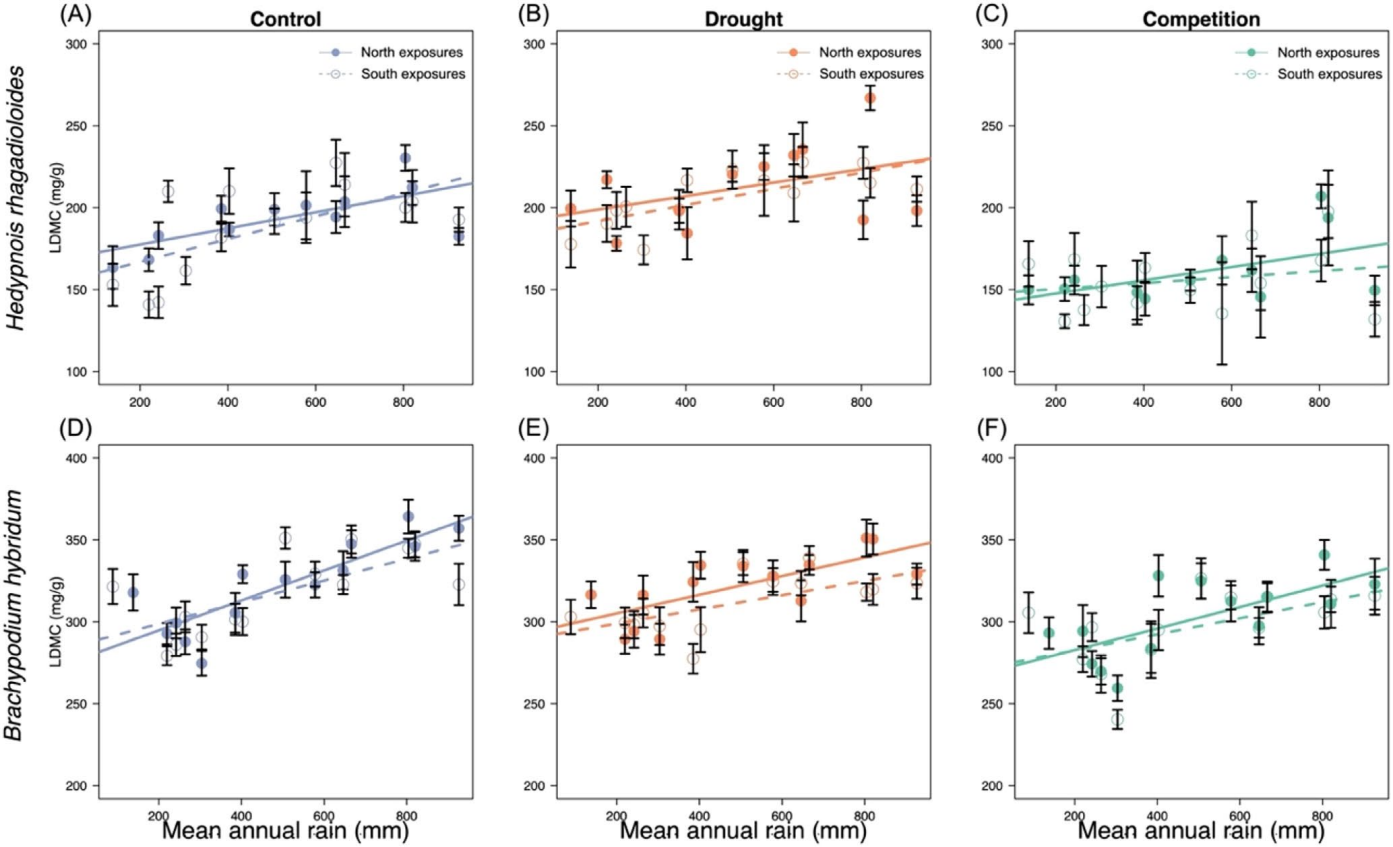
Leaf dry matter content (LDMC) in two plant species grown under three greenhouse treatments: Control (A, D; in blue), drought stress (B, E; in orange), and competition (C, F; in green). Plants originated from different sites across the macroclimatic rainfall gradient (*x*‐axes), and from either north‐facing (filled circles) or south‐facing exposures (open circles). Error bars represent ± SE of the mean. Statistical results are provided in Table 4.

Across the macroclimatic gradient, SLA decreased significantly in four of the five species (all but *Hippocrepis unisiliquosa* ) as rainfall increased (Table 1; Figure 3), indicating more conservative leaves in plants originating from wetter sites. Both *Hedypnois rhagadioloides* and *Brachypodium hybridum* repeated this pattern in the second experiment (Table 3; Figure 5), underlining that these changes had a genetic basis. LDMC changed significantly with rainfall by increasing toward wetter sites in *Hedypnois rhagadioloides* (in both experiments, Tables 2 and 4; Figures 4 and 6), in *Avena sterilis* (Table 2; Figure 4), and in *Brachypodium hybridum*, yet only in the second experiment (Tables 2 and 4; Figures 4 and 6). No significant change in LDMC with rainfall occurred in *Hippocrepis unisiliquosa* and *Anagallis arvensis* .

**TABLE 2:** Results of linear mixed models testing the response of LDMC to annual rainfall at origin along the macroclimatic gradient (rain), exposure (north vs. south) in five annual species. Degrees of freedom (df) were approximated via Kenward–Roger. Bold indicates significant *p*‐values (*p* < 0.05).

Species		Df, Df res	F	*p*
*Hedypnois rhagadioloides*	Rain	1, 10.8	24.79	**0.0004**
	Exposure	1, 310.9	0.29	0.5891
	Rain × Exposure	1, 311.7	0.81	0.3695
*Brachypodium hybridum*	Rain	1, 13.6	1.96	0.1839
	Exposure	1, 418.9	0.51	0.4756
	Rain × Exposure	1, 390.2	1.97	0.1608
*Avena sterilis*	Rain	1, 11.9	5.03	**0.0447**
	Exposure	1, 360.4	3.12	0.0783
	Rain × Exposure	1, 363.9	1.83	0.1772
*Hippocrepis unisiliquosa*	Rain	1, 14.0	0.10	0.7541
	Exposure	1, 319.7	9.05	**0.0028**
	Rain × Exposure	1, 128.5	2.20	0.1409
*Anagallis arvensis*	Rain	1, 12.3	1.03	0.3301
	Exposure	1, 106.5	0.48	0.4902
	Rain × Exposure	1, 108.7	0.004	0.9468

Exposure effects (microclimate) were generally less pronounced than macroclimatic rainfall effects, and when present, their direction varied among species. *Hippocrepis unisiliquosa* had significantly higher LDMC and slightly lower SLA on plants from south exposures, despite being the only species with no rainfall effect (Tables 1 and 2; Figure 3D, 4D). Conversely, and only in the second experiment, *Hedypnois rhagadioloides* had overall higher SLA and *Brachypodium hybridum* lower LDMC in plants originating from south exposures (Figures 5 and 6), which aligned with their patterns along the macroclimatic gradient.

Rain × Exposure interactions were rarely significant across species, indicating that the microclimatic effects of exposure were largely similar along the macroclimatic (rainfall) gradient. However, a weak interaction was observed for SLA in *Hedypnois rhagadioloides* (Table 3), indicating some exposure‐specific sensitivity.

**TABLE 3:** Results of linear mixed models testing the response of SLA to annual rainfall at origin along the macroclimatic gradient (rain), exposure (north vs. south), and treatment (control (c), drought (d), competition (cm)) in two annual species, *Hedypnois rhagadioloides* and *Brachypodium hybridum*. Bold indicates significant *p*‐values (*p* < 0.05).

	*Hedypnois rhagadioloides*				*Brachypodium hybridum*			
	Df, Df res	F	*p*	Post—hoc	Df, Df res	F	*p*	Post—hoc
Rain	1, 11.4	16.35	**0.00180**		1, 13.3	8.71	**0.01098**	
Exposure	1, 248.2	5.39	**0.02104**		1, 414.3	0.05	0.82852	
Treatment	2, 436.2	237.04	**< 0.0001**	cm > c > d	2, 802.2	205.45	**< 0.0001**	cm > c, d
Rain × Exposure	1, 254.3	3.97	**0.04752**		1, 400.0	0.37	0.54226	
Rain × Treatment	2, 443.6	3.50	**0.03108**	d > c	2, 798.3	4.15	**0.01601**	cm > c
Exposure × Treatment	2, 436.5	0.98	0.37560		2, 802.5	0.28	0.75777	
Rain × Exposure × Treatment	2, 443.3	2.38	0.09410		2, 797.8	1.18	0.30782	

Overall, macroclimatic origin influenced SLA and LDMC in most species (except *Hippocrepis unisiliquosa* ), leading to more conservative leaf traits (lower SLA and sometimes higher LDMC) in plants originating from wetter sites. Microclimatic exposure effects were weaker and significant for only three species, yet aligned in two species with the macroclimatic pattern, showing more conservative leaves in plants originating from south exposures. Whether macroclimatic and microclimatic origin affected SLA or LDMC was scattered independently across grass species (*Brachypodium hybridum, Avena sterilis*) and forb species (*Hedypnois rhagadioloides, Hippocrepis unisiliquosa*, *Anagallis arvensis* ).

### 3.2 Effects of Experimental Treatments on Leaf Traits

In the second experiment, both the forb *Hedypnois rhagadioloides* and the grass *Brachypodium hybridum* displayed strong plasticity to the experimental treatments, and both in largely similar ways. SLA differed significantly among treatments, being highest under competition (Figure 5F,C), lowest under drought (Figure 5E,B) and intermediate in the control (Figure 5D,A) in both species (Table 3). LDMC mirrored this pattern, with the lowest values under competition (Figure 6F,C), the highest under drought (Figure 6E,B) and intermediate values in the control (Figure 6D,A; Table 4).

**TABLE 4:** Results of linear mixed models testing the response of LDMC to annual rainfall at origin along the macroclimatic gradient (rain), exposure (north vs. south), and treatment (control (c), drought (d), competition (cm)) in two annual species, *Hedypnois rhagadioloides* and *Brachypodium hybridum*. Bold indicates significant *p*‐values (*p* < 0.05).

	*Hedypnois rhagadioloides*				*Brachypodium hybridum*			
	Df, Df res	F	*p*	Post Hoc	Df, Df res	F	*p*	Post hoc
Rain	1, 11.4	14.30	**0.00286**		1, 13.2	16.31	**0.00136**	
Exposure	1, 250.5	2.14	0.14485		1, 414.1	4.09	**0.04386**	
Treatment	2, 432.6	129.10	**< 0.0001**	d > c > cm	2, 797.9	47.53	**< 0.0001**	c, d > cm
Rain × Exposure	1, 256.8	2.10	0.15751		1, 408.4	2.10	0.14836	
Rain × Treatment	2, 440.3	3.60	**0.02837**	c > cm	2, 794.5	5.33	**0.00502**	c > d
Exposure × Treatment	2, 433.1	0.03	0.97387		2, 798.3	0.96	0.38349	
Rain × Exposure × Treatment	2, 440.1	0.86	0.42258		2, 794.1	0.13	0.87587	

Treatment effects were not uniform across the rainfall gradient, indicated by the significant Rain × Treatment interactions for SLA and LDMC in both species (Tables 3 and 4). The post hoc tests revealed that in the competition treatment compared to control, SLA increased stronger in mesic than in arid populations (*c*. +10 vs. +5) in *Brachypodium hybridum* (Figure 4D,F), and LDMC decreased stronger in mesic than arid populations (c. −50 vs. −20) in *Hedypnois rhagadioloides* (Figure 6A,C). In the drought treatment compared to control, SLA decreased stronger (i.e., more conservative leaves) in arid than in mesic populations (*c*. −5 vs. −1) in *Hedypnois rhagadioloides* (Figure 5A,B); LDMC increased in arid populations (*c*. +15, more conservative) but decreased in mesic populations (*c*. −30, more acquisitive) in *Brachypodium hybridum* (Figure 6D,E). These results indicate that competition‐related plasticity toward more acquisitive leaves was stronger in mesic populations, while drought‐related plasticity toward more conservative leaves was stronger (for SLA) or exclusively observed (for LDMC) in arid populations.

## 4 Discussion

### 4.1 Macroclimatic Trait Divergence and the Prevalence of Acquisitive Strategies in Annual Plants

This study examined how macro‐ (rainfall origin) and microclimatic (exposure aspect) gradients influence two leaf traits—SLA and LDMC—across five annual plant species under common greenhouse conditions isolating genetic variation among origins. Further, drought and competition treatments on two focal species assessed plasticity. Macroclimatic effects and plastic responses were largely consistent among species, whereas microclimatic effects were weak and more species‐specific.

We detected clear changes across the macroclimatic rainfall gradient: SLA increased with aridity in four of the five study species (except *Hippocrepis unisiliquosa* ; Figures 3 and 5), and LDMC declined in three species, *Hedypnois rhagadioloides*, *Avena sterilis* , and (second experiment only) *Brachypodium hybridum* (Figures 4 and 6). The shifts persisted in the second experiment (i.e., second generation), confirming that they had a genetic basis. As the shifts occurred in grass species and forb species, they indicate that more acquisitive leaves had repeatedly evolved in plants from drier sites, counter to the global LES, which predicts lower SLA and higher LDMC under drought (Reich 2014; Wright et al. 2004). While perennial species from arid sites commonly align with LES predictions and express more conservative leaves (Baruch et al. 2017; Carlson et al. 2016; Ramírez‐Valiente et al. 2010), our results suggest that in annuals, arid environments often favor acquisitive strategies; life‐history differences may thus alter adaptive trajectories.

In contrast to perennials, annuals do not tend to maintain long‐lived, stress‐tolerant tissues with low SLA and high LDMC to withstand drought (O'Hara et al. 2016; Kooyers 2015; González‐Paleo et al. 2019; Ning et al. 2022). Rather, numerous studies showed that annuals evolved shorter life cycles in drier sites in order to escape drought (Kigel et al. 2011; Kooyers 2015; Kurze et al. 2017; Metz et al. 2020; Gade and Metz 2024). Our results for five species suggest that evolving more acquisitive leaves (or at least maintaining similar SLA or LDMC) despite increasing macroclimatic aridity facilitates the fast growth needed for completing the life‐cycle faster in drier sites. They align with previous findings where annuals did not evolve more conservative leaves in drier sites: *Plantago patagonica* and *Brassica rapa* from Southwestern US evolved increased SLA and accelerated development in arid populations (Christie et al. 2022; O'Hara et al. 2016), and three Mediterranean annuals maintained at least similar SLA and LDMC across our rainfall gradient in Israel (Bergholz et al. 2017; Kurze et al. 2017). However, exceptions exist: annual *Chamaecrista fasciculata* from temperate central US (Etterson 2004) and *Arabidopsis thaliana* from temperate Europe (Fletcher et al. 2022) evolved more conservative leaves in drier sites, perhaps because winter cold in mesic sites added another constrain. Importantly, arid populations with fast life cycles—and acquisitive leaves—are not more drought tolerant during the growing season than their mesic counterparts (Gade and Metz 2024), highlighting that drought escape carries significant costs alongside its adaptive benefits.

### 4.2 Microclimatic Modulation of Leaf Traits: Weak and Species‐Specific

Microclimatic variation in SLA and LDMC due to exposure aspect was generally weak, often non‐signifcant, and differed between species. Across both experiments, exposure effects were often non‐significant and species‐dependent. In the first experiment, *Hippocrepis unisiliquosa* was the only of five species with significant exposure effects. Here, plants originating from south exposures showed more conservative, drought‐tolerant leaves with lower SLA and higher LDMC (Figures 3 and 4). Opposite responses were observed in the forb *Hedypnois rhagadioloides* and the grass *Brachypodium hybridum*, but only in the second experiment, perhaps due to larger sample size and additional treatments: *Hedypnois rhagadioloides* had higher SLA and *Brachypodium hybridum* lower LDMC on south exposures (Figures 5 and 6). These patterns reflect the acquisitive drought‐escape strategy that both species exhibited toward drier sites along the macroclimatic gradient, corroborating that they have adaptive value. Indeed, a parallel study analyzing our second experiment demonstrated microclimatic local adaptation between exposures, namely that south‐exposure plants were better adapted to drought in *Brachypodium hybridum* and north‐exposure plants better adapted to competition in *Hedypnois rhagadioloides* (Gade and Metz 2024). The microclimatic divergence in leaf traits revealed now in the same plants plausibly contributed to this adaptation, likely in combination with additional traits (Kurze et al. 2017).

Our overall weak and partly contrasting exposure effects on leaf traits match studies where leaf traits varied little between exposures within two Mediterranean shrub species (Blanco‐Sánchez et al. 2022), and a previous study on *Brachypodium hybridum* originating from six sites along the same macroclimatic gradient (Kurze et al. 2017). They corroborate that genetically based leaf trait divergence is often weak and insignificant without large sample sizes, although south exposures in dryland ecosystems are typically drier while north exposures harbor denser, more mesic vegetation (Figure 1; Kutiel and Lavee 1999; Nevo 2012; Yang et al. 2021; Blanco‐Sánchez et al. 2022). One reason for that is probably gene flow across the rather short distances between exposures that weakens microclimatic local adaptation (Kawecki and Ebert 2004; Savolainen et al. 2013). As the amount of gene flow varies with pollination and dispersal syndrome, the degree of microclimatic local adaptation can be species‐specific, and may explain why leaf traits differed in only three of our species between exposures. Moreover, selection favored drought escape via acquisitive leaf traits similarly on both exposures in two woody perennials (Blanco‐Sánchez et al. 2022; Carlucci et al. 2015), perhaps also because both exposures experience similar growing season lengths—in contrast to macroclimatic rainfall gradients where seasons are typically shorter in drier sites (Noy‐Meir 1973; Gade and Metz 2024). Understanding trait divergence and small‐scale adaptation between opposing exposures in the field (Pan et al. 2023; Zhou et al. 2023) thus needs to consider both genetic and plastic mechanisms.

### 4.3 Plasticity and Context‐Dependent Trait Shifts

We found strong and consistent plastic responses to drought and competition in both the grass *Brachypodium hybridum* and the forb *Hedypnois rhagadioloides*, demonstrating substantial intra‐individual flexibility in leaf trait expression. In both studied species, drought consistently reduced SLA and increased LDMC, whereas competition produced the opposite pattern, with higher SLA and lower LDMC (Figures 5 and 6). These responses align with LES theory (Reich 2014; Wright et al. 2004) and earlier studies: under water‐limited conditions, plastically reduced SLA and elevated LDMC offer a conservative, resource‐saving strategy that enhances structural integrity and reduces water loss (Etterson 2004; Kramp et al. 2022; Wheeler et al. 2023). Conversely, our results add scarce empirical evidence that under shading or competition, increased SLA and lower LDMC are adopted to facilitate light capture and carbon gain, reflecting an acquisitive strategy aimed at maximizing growth under biotic constraints (Lipowsky et al. 2015; Yang et al. 2021). Notably, the magnitude of plastic responses was similar to trait shifts along the rainfall gradient, while their direction ran counter to genetic divergence along the rainfall gradient.

Our findings elucidate how plasticity mediates trade‐offs among light acquisition, growth, and drought tolerance, consistent with the triangular trade‐off framework between drought stress, fast life‐cycles, and competition (Gade and Metz 2024) derived from Grime's CSR theory (Grime 1974, 1977). While leaf traits were often more acquisitive (instead of more conservative) in plants originating from drier sites to facilitate drought escape and rapid life‐cycles, experiencing drought stress caused plasticity initiating more conservative leaves to avoid desiccation; vice‐versa, experiencing competition amplified acquisitiveness in leaves to facilitate light capture and growth. These strategic shifts suggest that plasticity allows plants to balance conflicting demands—maximizing performance when water is plentiful but competition is high, while minimizing risk under drought stress. Such plasticity likely contributes to persist in ecosystems where drought stress and competition vary in space and time (Metz and Tielbörger 2016).

Interestingly, significant rain × treatment interactions (Tables 3 and 4) revealed that plasticity varied across the rainfall gradient: mesic populations exhibited stronger plasticity to competition and arid populations tended to express stronger plasticity to drought (Figures 5 and 6). Because competition is more intense in mesic sites of the gradient (Schiffers and Tielbörger 2006; Metz and Tielbörger 2016) and drought stress more severe in arid ones, these results suggest that plasticity has evolved in response to the dominant environmental pressures prevailing at each population's origin. Together, our findings highlight that plasticity enables rapid, context‐dependent adjustments of leaf traits, thereby optimizing resource use under both abiotic and biotic stress. Such environmentally targeted plasticity likely contributes to population persistence across the rainfall gradient, supporting the idea that local adaptation encompasses genotypes differing not only in baseline trait values but also in their capacity for plasticity (Ramírez‐Valiente et al. 2010; Oyanoghafo et al. 2023; Ramos‐Muñoz et al. 2024).

## 5 Conclusions

This study shows that annual plants frequently diverge from classical drought‐adaptive expectations across macro‐ and microclimatic aridity gradients. Instead of expressing conservative leaf traits, populations from drier habitats typically evolved higher SLA and lower LDMC, consistent with acquisitive, fast‐growth strategies that enable drought escape. By contrast, exposure aspect produced weaker and less consistent effects, likely reflecting weaker selection pressures at the microclimatic scale and the homogenizing influence of gene flow. Plasticity further modulated trait expression in origin‐specific ways. Drought generally reduced SLA and increased LDMC, while competition had the opposite effect. Such adjustments highlight trade‐offs between growth, light acquisition, and stress tolerance, consistent with CSR theory (Grime 1974, 1977), and illustrate how annuals adjust trait expression under fluctuating abiotic and biotic conditions. Together, these findings reinforce Gade and Metz's (2024) conclusion that local adaptation along aridity gradients is shaped not solely by drought tolerance, but also by life‐history timing and competitive interactions. Our results extend this view by showing that leaf trait variation and plasticity contribute to these patterns, with expression strongly dependent on macroclimatic rather than microclimatic origin. More broadly, our work underscores the importance of quantifying both genetic differentiation and plasticity across multiple environmental gradients to improve predictions of annual plant responses to future climatic shifts.

## Author Contributions

**Ruchi Tiwari:** conceptualization (equal), data curation (equal), formal analysis (equal), methodology (supporting), visualization (equal), writing – original draft (lead), writing – review and editing (equal). **Francine Hellmanzik:** methodology (equal). **Florian Gade:** methodology (equal). **Johannes Metz:** conceptualization (equal), data curation (equal), funding acquisition (lead), project administration (lead), supervision (lead), visualization (equal), writing – review and editing (equal).

## Funding

This work was supported by a FoKo starting grant of the University of Hildesheim to J.M. and by the Deutsche Forschungsgemeinschaft (DFG) grant 535094127 to J.M.

## Conflicts of Interest

The authors declare no conflicts of interest.

## Supporting information


**Table S1:** ece373277‐sup‐0001‐Table S1.docx.

## Data Availability

The data supporting our results is publicly available at https://data.goettingen‐research‐online.de via https://doi.org/10.25625/UQQIJY.
