# Supplementary material for: Leaf Economics and Local Adaptation: Genetic and Plastic Responses Within Mediterranean Annual Species to Macro‐ and Microclimatic Aridity Gradients
Source: Ecol Evol. 2026 Mar 17;16(3):e73277. doi: 10.1002/ece3.73277 (PMC13093557; doi:10.1002/ece3.73277)
Supplement: Supplementary file 1 — Table S1: ece373277‐sup‐0001‐Table S1.docx. [file ECE3-16-e73277-s001.docx]

**Supporting Information for article:**

**Leaf Economics and Local Adaptation: Genetic and plastic responses within Mediterranean Annual Species to Macro- and Microclimatic Aridity Gradients**

**Table S1:** Properties of the 15 sampling sites along the macroclimatic rainfall gradient in Israel. Site names refer to the nearest town. Mean annual precipitation (rain) for each sampling site was obtained from the nearest station of the Israel Meteorological Service (www.ims.gov.il), calculated across the period 1984-2020. The interannual variation of rainfall (CV rain; coefficient of variation in rainfall among individual years) increased toward drier sites. Mean annual temperature per site was obtained from the worldclim.org data base and showed no trend along the gradient. Rain, CV rain, and temperature describe the macroclimate and were not separately available for the microclimatic difference between north and south exposures. Altitude above sea level (a.s.l.) and inclination (i.e. steepness of the slope) were similar between exposures, while the vegetation was usually sparser at south exposures. The experiment included slightly variable numbers of genotypes per site – exposure combination of five target species—*Brachypodium hybridum*, *Avena sterilis*, *Hedypnois rhagadioloides*, *Hippocrepis unisiliquosa* and *Anagallis arvensis*—as the species were scarce or even absent (NA) in some combinations.

|  |  |  |  |  |  | no. of included genotypes | | | | |  |  |  |  |
| --- | --- | --- | --- | --- | --- | --- | --- | --- | --- | --- | --- | --- | --- | --- |
| site code | site | rain (mm/yr) | CV rain (%) | temperature (°C) | exposure | *Brachypodium hybridum* | *Avena sterilis* | *Hedypnois rhagadioloides* | *Hippocrepis unisiliquosa* | *Anagallis arvensis* | altitude (m  a.s.l.) | inclination (°) | GPS location | Vegetation |
| HR | Harashim | 926 | 26.0 | 17.8 | N | 13 | 16 | 15 | 13 | 1 | 669 | 22 | 32.9646N, 35.3287E | dense woodland (c.80% tree cover) of *Quercus calliprinos*, *Calicotome villosa*, *Rhamnus lycioides, Pistacia lentiscus* |
|  |  |  |  |  | S | 15 | 4 | 15 | NA | 3 | 750 | 20 | 32.9533N, 35.3269E | open woodland (c.30% tree cover, 30% shrub cover) of *Quercus calliprinos, Calicotome villosa, Pistacia lentiscus* |
| EY | Ein Yaacov | 820 | 26.6 | 18.9 | N | 12 | 10 | 12 | 2 | 4 | 480 | 13 | 33.0057N, 35.2394E | dense woodland (c.80% tree cover) of *Quercus calliprinos, Calicotome villosa* |
|  |  |  |  |  | S | 17 | 7 | 16 | 15 | 7 | 490 | 12 | 33.0067N, 35.2394E | dense shrubland (c.75% shrub cover) of *Calicotome villosa, Sarcopoterium spinosum, Cistus spp., Quercus calliprinos* |
| EL | Eilon | 804 | 25.2 | 19.8 | N | 11 | 12 | 3 | NA | 9 | 225 | 12 | 33.0688N, 35.2099E | woodland (c.60% tree cover) of *Quercus calliprinos, Calicotome villosa, Sarcopoterium spinosum, Salvia fruticosa* |
|  |  |  |  |  | S | 20 | 20 | 16 | 7 | 7 | 200 | 15 | 33.0697N, 35.2057E | open shrubland (c.20% shrub cover) of *Sarcopoterium spinosum* with single *Quercus calliprinos trees* (<10% cover) |
| RM | Ramot Menashe | 666 | 27.7 | 19.7 | N | 19 | 15 | 11 | NA | 6 | 158 | 15 | 32.5876N, 35.0613E | dense grassland (c.70% cover) of annual & perennial herbaceous vegetation, 10% shrub cover of *Calicotome villosa* |
|  |  |  |  |  | S | 20 | 15 | 7 | NA | 8 | 140 | 10 | 32.5885N, 35.0558E | similar to N-slope |
| GL | Gelad | 646 | 26.1 | 19.5 | N | 17 | 19 | 15 | NA | 12 | 230 | 15 | 32.5677N, 35.1003E | dense grassland (c.70% cover) of annual & perennial herbaceous vegetation, 10% shrub cover *Majorana syriaca*, *Calicotome villosa,* *Sarcopoterium spinosum* |
|  |  |  |  |  | S | 20 | 16 | 17 | 2 | 9 | 230 | 13 | 32.5703N, 35.0986E | similar to N-slope |
| MA | Mata | 578 | 29.3 | 18.1 | N | 17 | 15 | 9 | 16 | 6 | 606 | 17 | 31.7117N, 35.0691E | shrubland (c.70% shrub cover) of *Calicotome villosa, Sarcopoterium spinosum, Cistus spp., Quercus calliprinos*; herbaceous vegetation between shrubs |
|  |  |  |  |  | S | 20 | 15 | 5 | 14 | 2 | 610 | 17 | 31.7130N, 35.0665E | similar to N-slope, but only c.60% shrub cover |
| BJ | Bet Jimal | 506 | 30.7 | 19.8 | N | 16 | 15 | 16 | 12 | 4 | 335 | 19 | 31.7212N, 34.9735E | dense shrubland (c.70% shrub cover) of *Cistus spp*., *Quercus calliprinos, Salvia fruticosa, Pistacia lentiscus, Sarcopoterium spinosum* |
|  |  |  |  |  | S | 17 | 14 | 14 | 17 | 4 | 335 | 20 | 31.7232N, 34.9728E | shrubland (c.50% shrub cover) of *Sarcopoterium spinosum* |
| GU | Bet Guvrin | 403 | 31.4 | 19.8 | N | 11 | 15 | 10 | 4 | 7 | 302 | 14 | 31.6223N, 34.9009E | shrubland (c.50% shrub cover) of *Sarcopoterium spinosum, Pistacia lentiscus, Quercus calliprinos, Rhamnus lycioides* |
|  |  |  |  |  | S | 12 | 15 | 7 | 14 | 6 | 306 | 14 | 31.6238N, 34.9014E | similar to N-slope |
| AM | Amatziya | 385 | 27.5 | 19.6 | N | 12 | 15 | 12 | 14 | 5 | 329 | 19 | 31.5467N, 34.9041E | shrubland (c.40% shrub cover) of *Rhamnus lycioides*, *Pistacia lentiscus*, *Sarcopoterium spinosum*, *Quercus calliprinos* |
|  |  |  |  |  | S | 7 | 17 | 15 | 15 | 0 | 330 | 18 | 31.5480N, 34.9049E | open shrubland (c.10% shrub cover) of *Sarcopoterium spinosum* and single trees (*Quercus calliprinos, Rhamnus lycioides*); dense herbaceous vegetation between shrubs |
| LHV | Lahav | 304 | 29.5 | 18.9 | N | 16 | 16 | NA | 14 | NA | 428 | 17 | 31.3914N, 34.8622E | shrubland (c.70% shrub cover) of *Sarcopoterium spinosum*, *Euphorbia hierosolymitana*; dense herbaceous vegetation between shrubs |
|  |  |  |  |  | S | 15 | 15 | 12 | 15 | NA | 430 | 17 | 31.3925N, 34.8621E | shrubland (c.40% shrub cover) of *Sarcopoterium spinosum*, *Euphorbia hierosolymitana*; herbaceous vegetation between shrubs |
| LM | Lehavim | 264 | 32.7 | 19.1 | N | 15 | 14 | NA | 15 | NA | 340 | 20 | 31.3624N, 34.8290E | shrubland (50% shrub cover) of *Sarcopoterium spinosum*; herbaceous vegetation between shrubs |
|  |  |  |  |  | S | 15 | 12 | 12 | 15 | NA | 340 | 20 | 31.3636N, 34.8288E | shrubland (20% shrub cover) of *Sarcopoterium spinosum*; loose herbaceous vegetation between shrubs |
| BS | Beer Sheva | 242 | 31.0 | 18.9 | N | 14 | 18 | 15 | 16 | NA | 420 | 14 | 31.3037N, 34.8196E | open shrubland (c.10% shrub cover) of *Thymelaea hirsuta, Sarcopoterium spinosum*; sparse herbaceous vegetation |
|  |  |  |  |  | S | 15 | 16 | 11 | 16 | NA | 420 | 13 | 31.3066N, 34.8193E | open shrubland (c.5% shrub cover) of *Thymelaea hirsuta, Sarcopoterium spinosum*; sparse herbaceous vegetation |
| OM | Omer | 220 | 29.3 | 19.2 | N | 13 | 15 | 14 | 16 | 1 | 342 | 17 | 31.2653N, 34.8192E | open shrubland (c.10% shrub cover) of *Thymelaea hirsuta*, *Ballota undulata*, *Teucrium spp.*; mainly annuals between shrubs |
|  |  |  |  |  | S | 15 | 7 | 13 | 15 | 1 | 340 | 15 | 31.2641N, 34.8199E | open shrubland (c.5% shrub cover) of *Thymelaea hirsuta, Ballota undulata*; mainly annuals between shrubs |
| NV | Nevatim | 138 | 37.5 | 19.2 | N | 15 | NA | 13 | 15 | 3 | 381 | 15 | 31.2120N, 34.8840E | open shrubland (c.25% shrub cover) of *Echinops spp.*; mainly annuals between shrubs |
|  |  |  |  |  | S | NA | NA | 11 | 15 | 5 | 380 | 15 | 31.2126N, 34.8840E | open shrubland (c.10% shrub cover) of *Echinops spp.*; mainly annuals between shrubs |
| SB | Sde Boqer | 89 | 42.3 | 18.8 | N | NA | NA | NA | 14 | 3 | 470 | 15 | 30.8528N, 34.7647E | open shrubland (c. 5% shrub cover) with *Zygophyllum dumosum*, *Artemisia sieberi*, *Hammada scoparia*; sparse annual cover between shrubs |
|  |  |  |  |  | S | 14 | NA | NA | 15 | NA | 470 | 15 | 30.8537N, 34.7639E | open shrubland (<5% shrub cover) with *Zygophyllum dumosum, Artemisia sieberi, Hammada scoparia;* sparse annual cover between shrubs |
